# Predictors of desire to limit childbearing among reproductive age women in high fertility regions in Ethiopia. A multilevel mixed effect analysis

**DOI:** 10.1186/s12889-023-15952-w

**Published:** 2023-05-30

**Authors:** Wubshet Debebe Negash, Tadele Biresaw Belachew, Desale Bihonegn Asmamaw, Desalegn Anmut Bitew

**Affiliations:** 1 Department of Health Systems and Policy, Institute of Public Health, College of Medicine and Health Sciences, University of Gondar, Gondar, Ethiopia; 2 Department of Reproductive Health, Institute of Public Health, College of Medicine and Health Sciences, University of Gondar, Gondar, Ethiopia

**Keywords:** Desire to limit, Reproductive age women, Multilevel analysis, High fertility regions, Ethiopia

## Abstract

**Background:**

A high fertility rate can have a number of expensive consequences for developing nations, such as limiting economic growth, adversely impacting women and their children’s health, and reducing access to quality education, nutrition, and employment. The problem is more obvious in Ethipia’s high fertility regions. Therefore, this study aimed to assess predictors of desire to limit childbearing among reproductive age women in high fertility regions in Ethiopia.

**Methods:**

The analysis was based on secondary data using the 2016 Ethiopian Demographic and Health Survey. Stata version 14 software was used for analysis. A multi-level mixed-effect logistic regression analysis was fitted. Adjusted Odds Ratio at 95% confidence interval was used to show the strength and direction of the association. Statistical significance was declared at a *P-* value less than 0.05.

**Results:**

The overall desire to limit childbearing in high fertility regions in Ethiopia was 37.7% (95% CI: 36.28, 39.17). Age; 25–34 (AOR = 3.74; 95% CI: 2.97, 4.73), 35–49 years (AOR = 14; 95% CI: 10.85, 18.06), women education; Primary education (AOR = 0.73; 95% CI: 0.61, 0.88), secondary and higher (AOR = 0.29; 95% CI: 0.19, 0.43), from the community level variables Oromia National Regional state (AOR = 5.86; 95% CI: 2.82, 12.23), high proportion of community level poverity (AOR = 0.67; 95% CI: 0.45, 0.98), and high proportion of community level media exposure (AOR = 1.53; 95% CI: 1.07, 2.19) were statistically significant factors for desire to limit childbearing in high fertility regions of Ethiopia.

**Conclusion:**

Nearly four in ten women had the desire to limit childbearing in high fertility regions in Ethiopia. Thus, to fulfill the women’s desire to limit childbearing, Ministry of Health and health facilities are needed to increase financial support strategies and Family planning programs that enable pregnant women from poor households to use health services. In addition, increasing community level media exposure are important interventions.

## Background

Ranking Ethiopia as the world’s 12th and Africa’s 2nd most (115 million) populous country and nearly 80% of the population resides in the rural settings [1, 2]. Developing nations like Ethiopia may face expensive costs as a result of high fertility rates such as limit opportunities for economic growth, threaten the health of women and children, reduce acess to quality education, nutrition, and employment [3, 4].

Over half of all worldwide fertility is found in sub-Saharan Africa (SSA) [5]. The limit on birth affects fertility rates more than birth intervals and plays a major role in causing a fertility transition [6]. A lot of sub-Saharan Africa countries women are interested in limiting births in addition to spacing births, and many are on action [7]. In SSA, modern contraceptives and other family planning strategies are not frequently used, which leads to unintended pregnancies [8, 9]. Despite the fact that limiters outnumber spacers in several countries, very little research has been conducted on the group of women in sub-Saharan Africa who wish to limit (or halt) [10]. In sub-Saharan Africa, trends indicate that more women are choosing to limit rather than delay [7].

Despite the increase in the population growth is not a problem by itself, an imbalance with the available resources and developmental speed of the country is a problem [11, 12]. For optimal population growth in a specific country, limiting fertility is necessary [13]. This is because the increase in fertility has multiple negative consquences on the mother and child health. As evidences from the Ethiopian 2016 Demographic and Health Survey report, there were 412 deaths from pregnancy-related causes per 100,000 live births. Furthermore, one in every 48 women between the ages of 15 and 50 will die during pregnancy, childbirth, or within two months of childbirth (or a combination thereof) among 1,000 women of the exact age of 15 [14].

The Ethiopian government attempted to decrease birth rates from 7.7 children per woman in 1990 to approximately 4.0 in 2015 by providing clinical and community-based contraceptives. Although this effort is being made, there is a 36% prevalence of contraception use among married reproductive-age women [14]. According to the 2005 and 2011 EDHS, among married women in rural Ethiopia, preference for limiting decreased from 41.4 to 36.9% [15]. In the 2016 EDHS report, the average fertility rate was 2.3 children per woman of reproductive age [16, 17]. However, three regions; Afar, Somali and Oromia are the high fertility rate regions in Ethiopia with fertility rates above 5.0, a value that is higher than the rate of 4.6 in Ethiopia and 2.47 worldwide [16, 18].

It is a concern that Ethiopia continues to have a high fertility rate, resulting in a high population growth rate that makes improvements in living standards challenging. Hence, understanding the factors influencing women’s desire to limit childbearing will be crucial for high fertility regions in Ethiopia with population policies and implementation programs aimed at reducing fertility, which will help to develop effective fertility control strategies. One of the key factors impeding population growth directly is, the proportion of women who intend to limit childbearing, which indicates a segment of the population that could potentially have an unwanted birth that makes improving living standards difficult [19]. In order to address this issue identifying predictors of desire to limit fertility and developing result based policy had paramount importance [19, 20].

Information combining specific high fertility regions in Ethiopia are scarce to understand the magnitude and predictors of desire to limit childbearing among reproductive age women. A multilevel modelling will contribute to understand both the individual and the community level factors that predict desire to limit childbearing. Thus, this research intends to fill the gap by examining the magnitude and predictors of desire to limit childbearing in Oromia, Afar, and Somali regions in Ethiopia using a multilevel approach.

## Materials and methods

### Study setting

A cross-sectional study of Ethiopian Demographic and Health survey (EDHS) data was used for this study. The survey was conducted by the Central Statistical Agency (CSA) in collaboration with the Federal Ministry of Health (FMoH) and the Ethiopian Public Health Institute (EPHI). EDHS is a national representative sample conducted from January 18 to June 27, 2016. There are nine regional states in Ethiopia (Tigray, Afar, Amhara, Oromia, Benishangul Gumuz, Gambela, South Nation Nationalities and People Region (SNNPR), Harari, and Somali), and two administrative cities (Addis Ababa and Dire-Dawa), 611 Districts, and 15,000 Kebeles (small administrative units in Ethiopia). The health care system in Ethiopia is structured in a three-tier system: primary, secondary, and tertiary levels of care. The primary level of care including primary hospitals, health centers, and health posts), the secondary level of care is delivered by general hospitals and the tertiary level of health care is given by specialized hospitals [21].

### Data source sampling procedure and population

The 2007 Ethiopian population and housing census central statistical agency (CSA) was used as a sampling frame. The EDHS employs a two-stage stratified sampling technique. Which makes the data nationally representative [22]. Totally, 645 enumeration areas (202 urban and 443 rural) used for the survey. Urban and rural samples were stratified based on region. Then proportional allocation was made within each stratum. In order to determine how many residential units are present in each enumeration areas (EAs), household listing operations were implemented. To select households, the resulting lists of households was used as a sampling frame. A total of 28 households were randomly selected from each cluster. The interviews were conducted only with preselected households. The eligibility was included those reproductive age women who are the usual members of selected households and visitors who slept in the house the night before the survey [23].

**Fig. 1:**
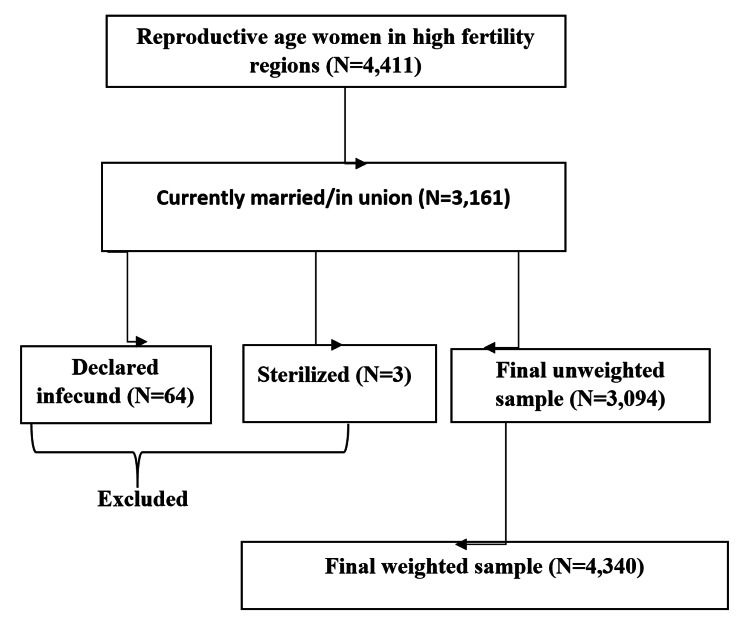
schematic presentation for desire to limit childbearing among reproductive age women in high fertility regions in Ethiopia, 2016

Source population included all women of reproductive age during the survey. For this study the study population includes all reproductive age women across all high fertility regions of Ethiopia. Accordingly, a total weighted sample of 4,340 reproductive age women were included in the study. Non married, infucund and sterilized women were excluded from this study (Fig. 1). We used the women’s recode (IR file) data set and extracted the dependent and independent variables. The data set is freely available and possible to download from the link: https://dhsprogram.com/data/available-datasets.cfm.

### Outcome variable

The outcome variable for this study was desire to limit childbearing among reproductive age women. The women were asked whether she wants to have another child at any time (soon, after two years) or wants no more children. Then it was categorized as ‘Yes = 1’ for those who desire to limit and ‘No = 0’ for those women who want a child within two years, after two years or those wants a child but not sure about the timing. As shown in Fig. 1, those women who were sterilized, declared infucund and unmarried were excluded from this study.

### Explanatory variables

Thirteen explanatory variables were considered and were grouped into individual and community level variables based on the availability in the EDHS dataset. These variables were selected based on their theoretical relevance and practical significance with desire to limit childbearing in previous studies [10, 24, 25].

### Individual level factors

The individual level factors were age, occupation, educational level, wealth index, media exposure, number of children, knowledge about family planning. Age was grouped as 15–24, 25–34, 35–49 years. Occupation was coded as working and not working. No formal education, primary education, secondary and higher education were the categories for highest educational level. Wealth index was recoded as poor, middle and rich. Frequency of reading newspapers/magazine, listening radio and watching television were each coded as not at all and less than once a week/at least once a week. Number of children were coded as 1–2, 3–6. Knowledge about family planning was coded as ‘yes’ for those women who knows about family planning and otherwise ‘no’.

### Community level factors

The community level variables were place of residence, region, community level education, community level poverty, community level media exposure, and distance to the health facility. Place of residence, region, distance to the health facility variables were based on their categorization in the DHS [6, 14, 26, 27]. The community level poverty, community level education and community level media exposure were generated by aggregating the individual level factors at cluster level and categorized them as high if the proportion is ≥ 50% and low if the proportion is < 50% based on the national median value since these were not normally distributed [28].

### Modeling approaches

A multilevel logistic regression model was used to identify the association between the individual and community level factors with desire to limit childbearing. Due to the hierarchical nature of EDHS data (individuals are nested within the communities), it is recommended that multilevel analyses be used to account for such data [29, 30]. A multilevel model also allows to track changes in variance across models as well as incorporate error terms at each level. Furthermore, the Likelihood Ratio (LR) test can be used to determine whether the standard logistic regression or the multilevel model fits the data best, in our case the LR-test was significant (p < 0.05). As a result, the LR-test indicates that the multilevel model is preferred over the flat model. This implies that If we use the standard logistic regression in the presence of significant LR, the result become biased and leads to wrong conclusion [31, 32].

STATA version 14 command “melogit” was used in fitting the models. All frequency distributions were weighted (v005/1,000,000) throughout the analysis to ensure that the EDHS sample was a representative sample and to obtain reliable estimates and standard errors before data analysis.

The first step was a graphical representation of the desire to limit childbearing among reproductive age women. The second step was a bivariable analysis that calculated the proportion of desire to limit childbearing across the independent variables with their *p*-values. All the variables having a p-value less than 0.2 in bivariable analysis were used for multivariable analysis. For the multivariable analysis, adjusted odds ratios with 95% confidence intervals and a p-value of less than 0.05 were used to identify predictors of desire to limit childbearing. In the final step of the analysis, a multilevel logistic regression analysis comprising fixed effects and random effects was done.

The results of the fixed effects of the model were presented as adjusted odds ratio (AOR) while the random effects were assessed with intra-class correlation coefficient (ICC) [33]. Four models were fitted; null model (model 0) which shows the variations in the desire to limit childbearing in the absence of any independent variables. Model I adjusted for the individual-level variables, Model II adjusted for the community level variables, and model III adjusted for both individual and community level variables [33, 34]. Simultaneously, model fitness was done using the deviance (-2 log likelihood). Variance inflation factor (VIF) was used to check for multicollinearity among independent variables and it was found no multicollinearity (mean value for the final model = 1.74).

### Ethics approval and informed consent

The ethical approval and permission to access the data were obtained from the DHS website www.measuredhs.com. All methods were approved by ICF International and an Institutional Review Board (IRB) in Ethiopia, in accordance with United states Department of Health and Human Services requirements for human subject protection. Ethical clearance was obtained by the Institutional Review Board of Demographic and Health Surveys (DHS) program data archivists after the consent manuscript was submitted to DHS Program/ICF International. Informed consent was obtained from all subjects and/or their legal guardian(s) of minors age below 16. No information obtained from the data set was disclosed to any third person. The study is not experimental study. Further explanation of how the DHS uses data and its ethical standards can be found at: http://goo.gl/ny8T6X.

## Result

### Descriptive results

**Fig. 2:**
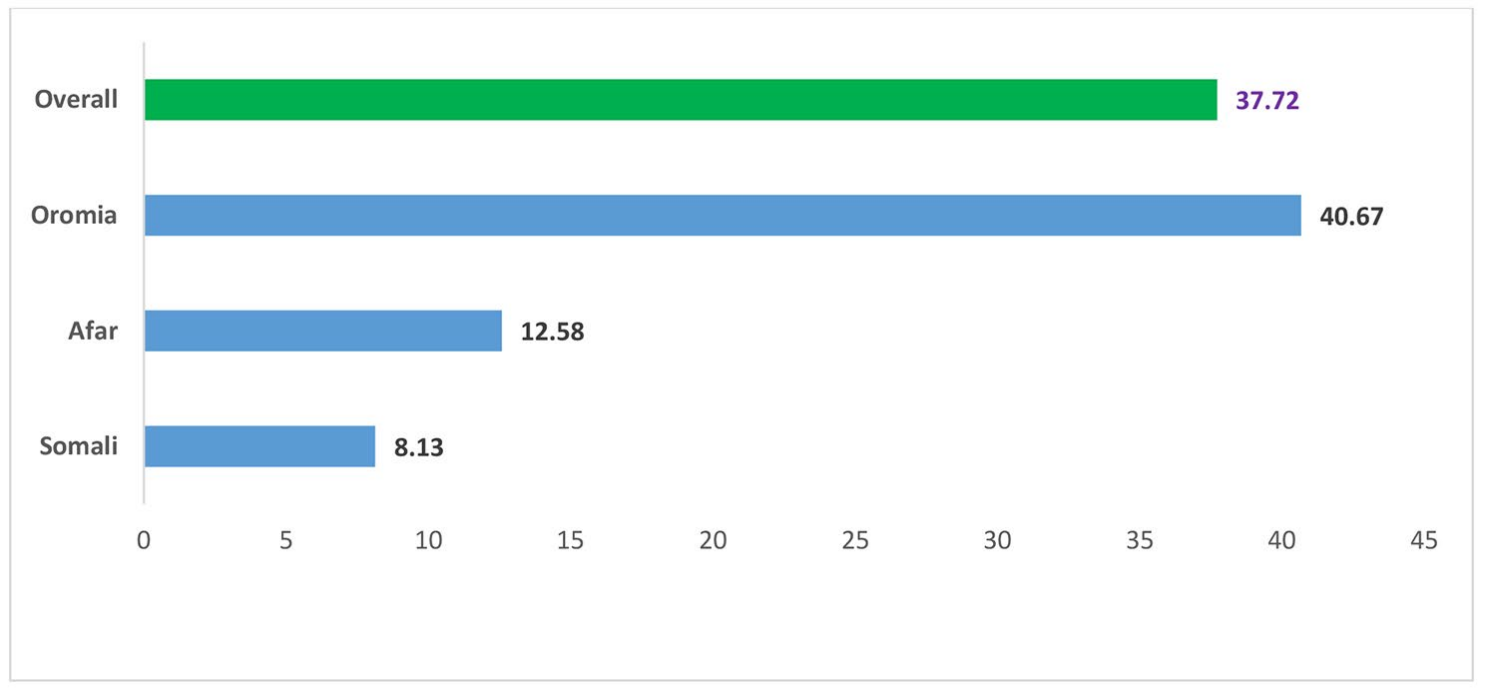
Prevalence of desire to limit childbearing among reproductive age womenin high fertility regions in Ethiopia

The overall magnitude of desire to limit childbearing in high fertility regions of Ethiopia was 37.72% (95% CI: 36.28, 39.17). Oromia region was accounted the highest (40.67%) desire to limit children (Fig. 2).

### Individual and community level characteristics of reproductive age women

In this study a total weighted sample of 4,340 reproductive age women were participated. The majority (44.6%) of the study participants were grouped under the age category of 25–34 years with a mean age of 29.64 ± 8.14 years. The majority (64.36%) of the women had no formal education. Most (70.88%) of the women perceived distance to the health facility as a big problem. Of the study participants 2764 (63.69%) were from community having high proportion of poor. With regard to community level media exposure, 2593 (59.74%) of the women were from communities having high proportion of media exposure (Table 1).

**Table 1:** Individual and community level characteristics of reproductive age women in high fertility regions in Ethiopia, 2016 (n = 4,340)

Variables	Categories	Frequency(n)	Percentage (%)
Age of women	15–24	1085	25.01
	25–34	1936	44.60
	≥ 35	1319	30.39
Household wealth index	Poor	1893	43.61
	Middle	848	19.54
	Rich	1599	36.85
Educational status of the women	No formal education	2792	64.33
	Primary	1232	28.40
	Secondary and higher	316	7.27
Occupation	Working	1744	40.18
	Not working	2596	59.82
Number of children in the household	No	997	22.98
	1–2	2825	65.10
	3 and above	518	11.92
Media exposure	Yes	1019	23.47
	No	3321	76.53
Distance to the health facility	Big problem	3076	70.88
	Not big problem	1264	29.12
Residence	Urban	476	10.97
	Rural	38.64	89.03
Region	Afar	854	27.60
	Somali	942	30.45
	Oromia	1298	41.95
Community level education	High	3144	72.44
	Low	1196	27.56
Community level poverty	High	2764	63.69
	Low	1576	36.31
Community level media exposure	High	2593	59.74
	Low	1747	40.26

### Random effects (measure of variations) results

The null model in the random effects, showed that a significant statistical differences in the odds of desire to limit childbearing with a community variance of 64.25%. Moreover, the intra-class correlation coefficient in the null model revealed that the 16.34% of the total variability of desire to limit childbearing accounted for differences between clusters. Additionally, the median odds ratio revealed that there was heterogeneity on desire to limit childbearing among different clusters. Accordingly, if we randomly choose an individual from two different clusters, those women from clusters having high prevalence had 1.54 times higher odds of being having desire to limit child bearing as compared to women from the lower prevalence of desire to limit cluster.

The result revealed that, there is a higher PCV of 0.6392 in the final model. Which is interpreted as 63.92% of the variations of desire to limit child bearing were attributable to both individual and community-level factors. With regard to model comparison, the third model was selected as a final model since it has the lowest (4606.44) deviance (Table 2).

**Table 2:** Multilevel analysis of factors associated with desire to limit childbearing in high fertility regions in Ethiopia, 2016 (n = 4,340)

Variables	Categories	Desire to limit childbearing		Model II AOR (95% CI)	Model III	Model IV
		Yes n(%)	No n(%)		AOR (95% CI)	AOR (95% CI)
Age in years	15–24	133(12.2)	953(87.8)	1		1
	25–34	687(35.5)	1249(64.5)	3.90(3.08, 4.93)		3.74(2.97, 4.73)*
	35–49	817(62.0)	502(38.0)	14.29(11.08,18.43)		13.99(10.85, 18.06)*
Women educational status	No formal education	1197(42.9)	1595(57.1)	1		1
	Primary	374(30.38)	858(69.62)	0.77(0.64,0.94)		0.73(0.61, 0.88)*
	Secondary and higher	66(20.69)	250(79.21)	0.32(0.21,0.47)		0.29(0.19, 0.43)*
Occupation of women	work	731(41.91)	1013(58.09)	1.21(1.03,1.42)		1.13(0.96,1.33)
	No work	906(34.90)	1690(65.10)	1		1
Wealth index	Poor	675(35.67)	1217(64.33)	1		1
	Middle	313(36.89)	535(63.11)	0.67(0.54,0.83)		0.58(0.47, 1.02)
	Rich	649(40.57)	950(59.43)	0.84(0.68, 1.05)		0.68(0.54, 1.15)
Media exposure	Yes	379(37.23)	640(62.77)	1.05(0.85,1.29)		0.99(0.81, 1.22)
	No	1258(37.87)	2064(62.13)	1		1
Number of children	0	429(43.06)	568(56.94)	1		1
	1 2	1026(36.31)	1800(63.69)	1.09(0.92,1.32)		1.11(0.91,1.34)
	3 6	182(35.11)	336(64.89)	1.08(0.82,1.43)	1	1.23(0.92,1.63)
Knowledge of family planning	yes	1622(38.32)	2611(61.68)	3.69(1.95,6.95)	2.45(1.62,3.69)	1.79(0.93,3.47)
	No	15(14.1)	93(85.9)	1	1	1
Residence	Urban	159(33.44)	317(66.56)			1
	Rural	1478(38.24)	2386(61.76)		1.56(1.02,2.39)	0.96(0.57,1.63)
Region	Afar	12(12.58)	82(87.42)		1	1
	Somali	25(8.13)	287(91.87)		0.70(0.32,1.52)	0.58(0.25,1.34)
	Oromia	1600(40.67)	2334(59.33)		4.69(2.42,9.12)	5.86(2.82, 12.23)*
Distance to the health facility	Not big problem	482(38.15)	782(61.85)		1.02(0.86,1.21)	1.02(0.84,1.23)
	Big problem	1155(37.54)	1921(62.46)		1	1
Community level education	High	1208(38.41)	1936(61.59)		0.84(0.63,1.13)	0.96(0.67,1.37)
	Low	429(35.89)	767(64.11)		1	1
Community level poverty	High	940(34.01)	1824(65.99)		0.72(0.53,0.98)	0.67(0.45, 0.98)*
	Low	670(44.22)	879(55.78)		1	1
Community level media exposure	High	1115(43.01)	1478(56.99)		1.48(1.11,1.99)	1.53(1.07, 2.19)*
	Low	522(29.86)	1226(70.14)		1	1
Random effect	Null model					
Variance		64.25		85.9	37.04	23.08
ICC (%)		16.34		20.7	10.12	6.56
MOR		2.12		2.40	1.75	1.54
PCV		*Ref*		62.90	63.67	63.92
Model comparission						
Deviance(2loglikelihood)		5517.64		4742.78	5383.24	4606.44
Mean VIF		….		1.36	1.61	1.74

*P-value < 0.05, ICC: Intra class corrolation cofficent; MOR: Median odds ratio; PCV: Proportional change in variance; AOR: adjusted odds ratio; CI: confidence interval; VIF: Variance Inflation Factor

### Fixed effects (measure of associations) results

In the final model, after adjusting for the individual and community level variables, age of the women, education of the women, wealth index, from the individual level variables, and region, community level poverty, community level media exposure from the community level variables were significantly associated factors with desire to limit .

Accordingly, the odds of desire to limit childbearing was 3.74(AOR = 3.74; 95% CI: 2.97, 4.73) times and 14 (AOR = 14; 95% CI: 10.85, 18.06) times higher among 25–34 and 35–49 years, reproductive age women, respectively as compared with women aged 15–24 years. The desire to limit childbearing had 27% less odds among reproductive age women who had primary education (AOR = 0.73; 95% CI: 0.61, 0.88), and 71% less odds among women who had secondary and higher education (AOR = 0.29; 95% CI: 0.19, 0.43) as compared with reproductive aged women of no formal education.

With regard to community level variables; the odds of desire to limit childbearing in Oromia National Regional State was nearly 6 (AOR = 5.86; 95% CI: 2.82, 12.23) times higher as compared to Afar National Regional state. Desire to limit childbearing was 33% (AOR = 0.67; 95% CI: 0.45, 0.98) less odds among reproductive age women from high community level poverty as compared to their counterparts. The odds of desire to limit childbearing among women from high community level media exposure was 1.53 (AOR = 1.53; 95% CI: 1.07, 2.19) times higher as compared to reproductive age women who had from low community level media exposure **(**Table 2**).**

## Discussion

The study attempted to assess the magnitude and associated factors of desire to limit childbearing among reproductive age women in high fertility regions in Ethiopia. The findings of our study will help policymakers and health facilities to develop tailored intervention strategies by considering the level of desire to limit childbearing and factors associated with desire to limit childbearing in high fertility regions in Ethiopia.

According to this study, nearly four in ten reproductive age women, 37.7% (95% CI: 36.28, 39.17), had desire to limit childbearing in high fertility regions in Ethiopia. The finding is lower than another previous study conducted in Ethiopia where 34.5% of the reproductive age women wanted to have no more children [35]. The discrepancy might be because of the former study was not excluded those reproductive age women who were declared infucund and sterilized, as compared to the current study. Moreover the former study was conducted in all Ethiopian regions in contrast to the current high fertility regions.

The study is lower than what was found in Ethiopia, where 43% of the reproductive age women wanted to have no more children. The difference might be accounted due to differences in the study period. The previous study was conducted based on the 2011 Ethiopian demographic health survey [36]. Similarly, the finding is lower than studies conducted in Pakistan which ranged from 44 to 47% [37, 38]. The possible justification for the variation might be the differences in sample size, source population and study setting. For this study we conducted a secondary data analysis and from the high fertility regions in Ethiopia, while the study conducted in Pakistan was primary data source and small sample size.

The finding is higher than study conducted in Nairobi, Kenya (33%), [39]. The possible justification for the difference might be difference in study design, study subjects. The study conducted in Nairobi was a longitudinal design among postpartum women whereas, the current study was conducted among all reproductive age women.

Accordingly, the odds of desire to limit childbearing was higher among women of age 25–34 and 35–49 years as compared with women aged 15–24 years. The finding is inline with studies in Ethiopia [25, 40], Bangladish [24], Malawi [41], and Egypt [26]. The possible justification for the higher probablity of desire to limit childbearing among older women might be due to the fact that the older women may have more children and prefer to limit or space the number of pregnancies than younger women with no or few children [36].

The desire to limit childbearing was less odds among reproductive age women who had educated as compared with reproductive aged women of no formal education. This is in line with another studies in Ethiopia [27, 36, 42]. The less desire to limit childbearing among educated women might be those women who had stayed in education may not have enough time to bearing child or they begin to have a children lately, to compensate this delay they begin to have less desire to limit childbearing [27, 43]. Moreover, those women who stayed in education may not have enough number of children at the end of their education which may lead them higher intention to have a child [36]. However, the finding is opposite as compared with another previous study conducted in Ethiopia, which states that the more educated women the less desire to have children [40]. Further studies might be required to understand how education correlated with the desire for more children.

With regard to community level variables; the odds of desire to limit childbearing in Oromia National Regional State was higher as compared to Afar National Regional state. This is in line with the EDHS 2016 report which states that the highest desire to limit is in Oromia region and Southern, Nations, Nationalities and people’s region, whereas the lowest desire is in Afar and Somali region [16]. This is possibly due to the reason that almost all of the Somali and Afar regions are Islam in religion. In which their book of Quran states as “Bring a lot of children for the prosperity of Islam” and contraception such as condoms, implants and pills are prohibited by religion [35, 44].

Desire to limit childbearing was less odds among reproductive age women from high proportion of community level poverty as compared to their counterparts. The finding is in line with studies conducted in Ethiopia [36, 42, 45]. Possible justification might be that in Ethiopian context the children are considered as an economic value who pay back during the old age [36]. The higher desire of children in Ethiopia might be also accounted due to the high proportion of rural resident agrarian population, in which enables them labor opportunities in agriculture. In Ethiopian culture, it is expected that when a child grows older, he/she will be a responsibility to support the parents for his/her upbringing. Additionally, couples (or parents) who place a high value on having children will seek out larger families who will later be able to take on the role of caregiving for their parents when they are older [36].

The odds of desire to limit childbearing among women from high proportion of community level media exposure was higher as compared to reproductive age women who had low community level media exposure. The finding is similar with studies conducted in Ethiopia and Uganda [42, 46]. The possible justification for desire to limit childbearing among media exposed women might be a large amount of information about fertility health, contraception use, and family planning programs has been provided by the public media. This can directly affect their desire to limit fertility [47, 48].

### Clinical and public health implications

In conclusive limiting fertility is necessary but not sufficient condition to bring down actual fertility, addressing other important factors such as unmet need might be very important. Fulfilling reproductive intention through providing informed choice to women, they can have the number of children they desire, which in the long run improves both their health and well-being, and ultimately affects macro-level health and development indicators. Facilitating the ability of women and couples to make informed choices about their desire of childbearing is also a fundamental human right that should carry on at the core of family planning programs.

### Strengths and limitations

The main strengths of this study were the use of regionally representative data, with a large sample size and the availability of individual and community-level factors. This study also used a multilevel-modeling technique to identify a more valid result that considers the survey data’s hierarchical nature. Despite these strengths, it has limitations due to the cross-sectional nature of the EDHS data. It does not show a temporal relationship between independent variables and the outcome variable. Rather than a simple yes or no question, latent variables such as knowledge about family planning must be measured by a composite of more than one questions.

## Conclusion

Nearly four in ten women had the desire to limit childbearing in high fertility regions in Ethiopia. Thus, to fulfill the women’s desire to limit childbearing, Ministry of Health and health facilities are needed to increase financial support strategies and Family planning programs that enable pregnant women from poor households to use health services. In addition, increasing community level media exposure are important interventions. Limiting the number of births is the only way to achieve desired fertility; this requires adapting sexual behavior and access to contraceptives. Desire to limit fertility also an indirect indicator for contraceptive use. By examining barriers from the supply-and demand-sides, family planning programs can meet this demand. Providing services and support to women in high fertility regions who wish to limit births is essential, as they are a distinctive audience that has long been unnoticed.

## Data Availability

The data set used for this study is freely available and possible to download from the link: https://dhsprogram.com/data/available-datasets.cfm.

## Abbreviations

AOR: Adjusted Odds Ratio
CSA: Central Stastical Agency
DHS: Demographic and Health Survey
EA: Enumeration Area
EDHS: Ethiopian demographic health survey
FMoH: Federal Ministry of Health
ICC: Intraclass Correlation
MOR: Median Odds Ratio
PCV: Proportional Change in Variance
VIF: Variance Inflation Factor
SSA: Sub-Saharan Africa
